# Study of power quality issues without and with Microgrid in Egypt

**DOI:** 10.1038/s41598-025-02297-0

**Published:** 2025-05-25

**Authors:** Montaser Abdelsattar, Mohamed M. Aly, Salah Saber Abu-Elwfa

**Affiliations:** 1 Electrical Engineering Department, Faculty of Engineering, South Valley University, Qena, 83523 Egypt; 2 Electrical Engineering Department, Faculty of Engineering, Aswan University, Aswan, 81542 Egypt

**Keywords:** Renewable energy sources, Power quality issues, Harmonic filter, Harmonic distortion, Voltage issue, Engineering, Electrical and electronic engineering

## Abstract

In comparison to power plants that depend on fossil fuels for electricity generation, Renewable Energy Sources (RES) provide more practicality and economic benefits. Achieving an optimal power balance between energy sources and demand poses significant challenges due to the inherent unpredictability and fluctuating loads associated with RES, like solar and wind energy. The feasibility of combining small-scale distributed power sources for generating electrical power at the distribution voltage level is generally recognized in the field of Microgrids (MGs). Certain energy generated from these sources is not readily usable due to its inherent characteristics. The effective management of power quality concerns poses a substantial technological obstacle when managing and operating MG systems, regardless of their connection to the primary electrical grid or their autonomous operation. The main factors contributing to these significant difficulties emerge from the design, operating strategy, inherent properties, and effectiveness MG systems’ dispersed energy sources. Power quality issues have arisen due to the notable rise nonlinear loads, uneven loads, and scattered generators. These problems include power losses, voltage decline and swelling, fluctuation, imbalance, and harmonics of electrical current and voltage. Energy quality can be improved in many ways, including the use of RES, connecting them to the electrical grid, and studying the extent to which energy quality improves before and after adding these sources. The importance of this study lies in knowing the issues of power quality and ways to improve it, as well as the impact of RES on the grid and ways to avoid these issues to obtain clean energy with high quality.

## Introduction

Renewable Energy Sources (RES) are energy sources that are both environmentally friendly and have little negative environmental effects. They also generate minimal secondary wastes and are sustainable in satisfying present and future financial and societal requirements^1^. Traditional power stations are commonly characterized by their substantial size and centralized nature. RES enables localized energy generation, which offers economic benefits and is a promising move toward sustainability, unlike traditional energy production methods^2^. A conventional grid necessitates a transportation and distribution infrastructure for the dissemination of the produced electricity. The distribution and transmission system incurs huge loss. There is an increasing inclination towards energy distribution and generating^3^, involves placing transformation of energy equipment in closer proximity to electricity users and substituting large modules for lower versions. It offers myriad advantages regarding technology, finances, and the environment^4^ (i) decreased transmission losses, (ii) enhanced voltage distribution, (iii) heightened overall effectiveness. The technological advantages include enhanced system stability and reliability. The economic advantages encompass decreased operational and maintenance expenses, along with enhanced productivity. Power quality issues present significant technical difficulties in the running and administration for the connected-to-the-grid and islanded MG systems^4^.

A Microgrids (MGs) is a network of electrical systems that includes both traditional and RES it supplies power to several small, distributed loads^5^. The operational characteristics and nature of supply of all sources are predominantly determined by the load they are connected to, despite the fact that they are all fundamentally electrical sources. Consequently, the entire system can exhibit very nonlinear operational characteristics. Although not a significant worry when many Distributed Generators (DGs) are connected to network, transitioning the MG from being connected to the network to operating in isolated (islanded) mode, or vice versa, does create certain power quality along with dependability concerns^6^. The utilization of Distributed Energy Resources (DERs) is guided by the principles of economy and energy efficiency, ensuring conformance to technical and environmentally sustainable standards^7^. The synergistic collaboration between DERs and Information Communication Technologies (ICT) allows for the harmonization of various infrastructures, promoting smart grid progress in both theoretical and practical realms^8^. Benefits influence to the maintenance of high power quality and the preservation of reliability. The concept of MG is introduced as a fundamental component of future smart grids^9^. Power quality has become a prominent concern in the field of smart grid technology^10^. Power quality refers to the no stationary disturbances that significantly disrupt the functioning of electrical equipment. Improper power supply can lead to malfunction, early failure, or complete inoperability of electrical devices or loads. Consequently, power quality analysis garners significant interest from scholars across multiple disciplines in this thought-provoking topic^11^. The power quality events that mostly occur in a distribution system can be classed as gradual voltage fluctuations, momentary voltage drops, rapid voltage fluctuations, harmonic distortions, and switching transients^12^. The prevalent power quality problems encountered in Alternating Current (AC) MG systems include voltage sags/swells resulting from abrupt changes in load, interruptions caused by the transition from grid-connected to alone mode, flicker, electrical reactive power, and harmonics generated throughout the process of conversion between AC and Direct Current (DC) systems. DC systems are seen as preferable to AC systems in terms of power quality concerns because harmonics and reactive power do not impact DC MGs^13^. This study shows cases of the enhancement of power quality by simulating and giving some following results as power factor, loss of power, Total Harmonic Distortion (THD), voltage swings, and voltage swelling^13^.

### The contributions of this paper as follows:

- Providing a contribution to review the issues of energy quality and how it can be improved by using renewable energy and also reviews some of the issues of using these sources. These solutions can be applied to different places and different applications.
- Utilization of renewable distributed energy resources.
- his work has been applied in one of the hotels in Hurghada, Egypt, in which the electrical loads were studied accurately and its behavior was studied, and the existing energy issues in the hotel were studied, and the study ended in improving the quality of energy using renewable energy sources, and this system can be applied anywhere, provided that the place and available spaces are studied to place solar panels and wind turbines and study wind speed and solar radiation to see whether this system is suitable or not.

Finally, power quality is determined by a variety of things. The system’s behavior under different operational settings is especially important. However, the resulting level of power quality is also determined by the system’s essential component properties, such as the characteristics of generating sets, including their control and load capabilities. Nonetheless, the quantity of harmonic distortion is mostly determined by the architecture and power of nonlinear loads, as well as the RES and equipment. Voltage imbalance in systems is typically determined by load imbalance (generally due to load failure or malfunction) and the number of DERs operating in parallel. The proposed model investigates load flow analysis and harmonic load flow for the MG before and after the addition of renewable energy sources, lowering the peak-to-average power ratio, minimizing distribution losses, improving the voltage profile, and minimizing harmonic distortion.

The structure of the paper is as follows: "Power Quality Issues In Microgrid" explains the power quality issue in MG, "Simulation results" is brief about harmonic filters, Section IV shows system design and components, "Summary" explains simulation results, conclusion at "Conclusion" limitation and future work at "Limitation and future work".

### Power quality issues in microgrid

Power quality issues will become more significant in future hybrid MGs as the penetration of single-phase/unbalanced loads, non-linear loads, and single-phase/unbalanced distribution units increase. Typically, the power quality problems encountered in the DC MG consist of variations in voltage and harmonic distortions. Within the AC MG, power quality concerns mostly revolve around voltage changes, unbalances, and harmonics^14^. Power quality of a system is determined according to various international standards. As per the IEC 61,000 standard, power quality refers to the specific attributes of electricity at a particular location within an electrical system, which are assessed based on a predefined set of technical criteria^14^. A MG has the capability of working either in isolated mode or grid-tied mode. Regardless of the scenario, it meets a power quality issue that varies between the two modes. AC MGs and DC MGs have arisen as alternative options in literature^15^. The DC MG exhibits characteristics that effectively mitigate numerous power quality issues. Nevertheless, the DC MG encounters distinct power quality challenges^15^. It is crucial to tackle challenges individually, as each system presents distinct problems that necessitate specific measures to alleviate them^15^.

#### Issues in AC microgrid

An AC MG is a compressed network that connects several scattered generators and various types of loads. The rapid changes in output and demand make it difficult to ensure system stability and maintain power balance. AC MG are associated with various difficulties. The key concerns are voltage stability, system reliability, and power quality^16^. In terms of power quality, MGs can operate in two modes: grid-connected and islanded. Power quality is expected to suffer detrimental consequences not only when operating in separation but also during the transitions from isolated and connected activities with the grid. These transitions can lead certain DER units to shift from volt-controlled mode (when operating in isolation) to current-controlled mode (when connected to the grid), resulting in voltage stability difficulties because of the delay in detecting inadvertent islanding^17–19^.

The power quality issues in AC MGs can be classified into the following categories:

#### Imbalance in power

An asymmetry of power emerges when a MG shifts from being interconnected with the primary power grid to functioning autonomously. When connected to a MG, an alternate micro power station operates independently from the main power grid to supply electricity. Devices that store energy are utilized for decreasing imbalances that occur at the period of the transition phase in MG because of the sluggish dynamic response of these power plants. Preserving phase sequence and magnitude of the voltage is crucial for achieving synchronization with the grid when normal mode is restored^16^.

#### Low voltage stability

Given the lower size of distributed generation and MG power distribution, the amount of assistance is less in comparison to the bigger grid. Consequently, there is an issue about the stability of low voltage. Power converters are employed for the purpose of controlling DGs and MG. Nevertheless, these converters possess limited power transfer capabilities compared to traditional power stations. At periods of heavy use, these converters promote a decrease in demand in the power network in comparison to traditional power plants^20^.

#### Voltage sag/swell

When a MG experiences a decrease in voltage, several issues can arise while using power electronics converters for distributed generating. During a sag event, the grid-connected load requires reactive power and attempts to deliver it; a converter employing power electronics technology is used. Distributed generators experience an overabundance of electric current in one or several phases. A further problem occurs when the maximum voltage threshold is surpassed, leading to the generator shutting down and causing a subsequent power outage^21^.

##### Issues in DC microgrid

While AC MG systems are frequently discussed in articles on power quality issues, there is a lack of emphasis on studying power quality issues in DC MG. The DC MG works in a grid-connected mode, which allows it to consume and provide electricity to and from the grid. It also functions in an isolated mode. Voltage imbalance in the bipolar DC bus, voltage transients in the AC grid, inrush current, and harmonics in the DC MG are examples of common power quality issues in DC MG^22–24^. There is now a lot of research being done on DC MG to better the integration of DER devices like energy storage and nonlinear loads. This is accomplished by minimizing the need for rectification, power inversion, or conversion phases. The power quality difficulties in DC MG come from variations between the purportedly steady voltage in a DC system and the actual voltage produced by power electronic converters^25^.

#### Voltage transient

AC MGs often face voltage transients, which have a comparable effect on DC MGs. Voltage transients mostly arise from the capacitor bank switching, the activation and deactivation of distributed generation integrated into a DC MG, and variations in the load. When a capacitor bank switches, a quick voltage shift is sent from the low-voltage AC side to the DC side of a MG via a rectifier. Studies have shown that the transient voltage can outperform the operational voltage by up to 194%^26^. Furthermore, it has been discovered that after this transitory phase, the voltage will stabilize at a greater level than before, which is roughly 111% of the operational voltage^26^.

#### Harmonics in DC microgrid

The absence of an AC-DC converter in the DC MG means that the system has no lower order harmonics. Nonetheless, greater use of a DC-DC converter would cause problems with electromagnetic interference. Despite current disagreements, the DC system is universally MG as harmonic-free. Harmonics are many frequencies that occur alongside the fundamental frequency, which is the principal frequency employed in power electronic converters. In a DC system, harmonics are the fluctuating voltage and current induced by the device’s operational frequency^27^. A DC MG system uses a number of Pulse Width Modulation (PWM)-based converters to supply power to various loads and producing stations. Capacitors are linked at both ends of the converter. The presence of impedance in the DC bus and numerous capacitors will cause the appearance of many resonance frequencies^27^.

#### Inrush current

In AC systems, inrush current is caused by strong inductive loads such as transformers and induction motors. However, in DC MGs, the cause of inrush current is different^22^. Harmonics are produced when power electronics converters connect various loads, scattered-producing stations, and varied storage devices. Therefore, appropriate filters are positioned on the side where the load is connected. Moreover, the AC side incorporates Electromagnetic-Interference (EMI) filters. When this EMI filter’s capacitor is first connected to the DC MG, it shows a noticeable current surge. The DC system’s voltage level, the capacitor’s impedance, and the converter’s capacitance all affect how much inrush current occurs^23^. In a DC MG, turning on a de-energized load is another way to induce inrush current. These loads demand a significant amount of inrush current due to the huge filter capacitors they contain. The inrush current’s magnitude is high enough to cause actual melting at the point of contact^27^.

#### Fault current

Three potential sources of fault current in a DC MG are the EMI filter capacitor, various distributed producing stations, and the capacitors connected to the grid through converters. The fault current is restricted by the converters’ rating because all power electronic converters are controlled in a closed-loop manner. Because of the power electronics converter, protection devices have trouble telling the difference between an overload situation and a fault state when there is a restricted fault current coming from the DC MG. As a result, designing a suitable protection system for a DC MG is very challenging^28^.

##### Power quality concerns related to demand side management

Four entities are involved in the main power quality issues that result from the use of Demand-Side Management (DSM) techniques in smart grids^29^. These entities are as follows: (1) When loads are connected or disconnected, the grid’s characteristics change, increasing harmonic levels and voltage variations; (2) When loads are reconnectable based on DSM, the load takes a long time to recover, increasing harmonic emission levels; (3) Sensitive equipment trips more frequently as a result of the frequent switching of DSM-enabled loads; (4) Economic pricing may encourage the simultaneous switching of several loads, resulting in transitory situations^30,31^.

### Harmonic filters

**Fig. 1:**
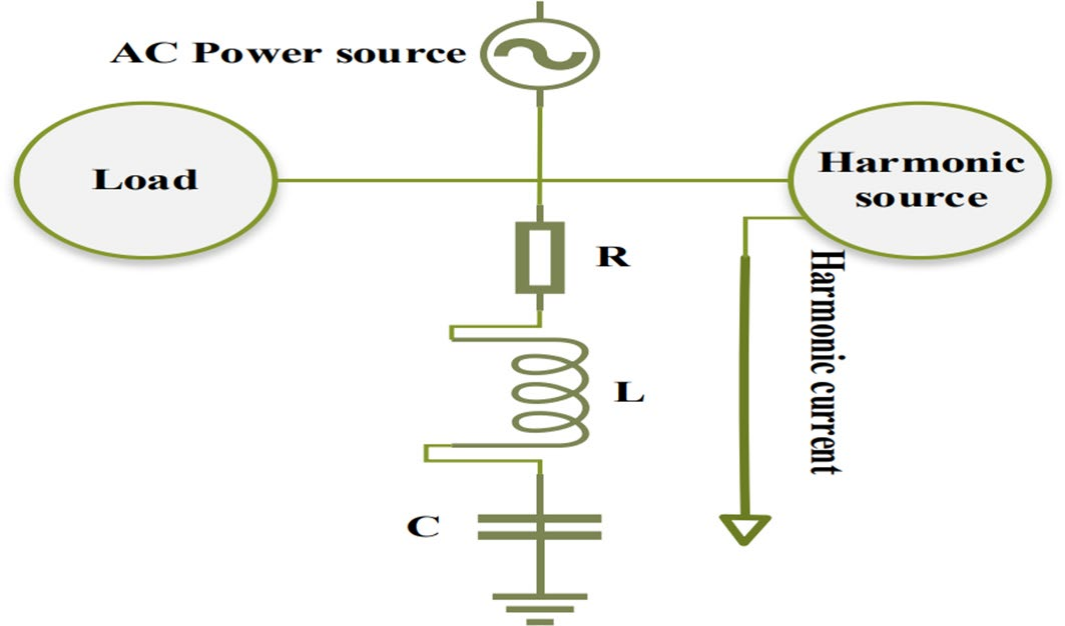
Basic principle of single-tuned filter^32^.

**Fig. 2:**
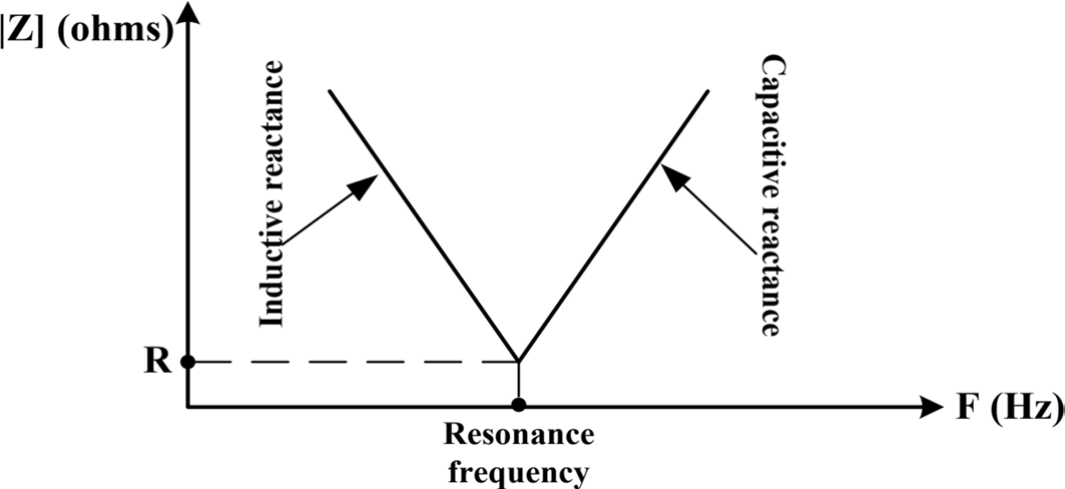
Single-tuned filter impedance characteristic^33^.

In electrical power systems, a single-tuned passive filter is a series resistance-inductive-capacitive circuit that is tuned to resonate at a certain frequency to provide harmonic suppression and mitigate system harmonic distortion levels^32^. To lessen harmonic propagation throughout the system, as seen in Fig. 1, the filter absorbs the harmonic currents injected by harmonic sources at a designated tuned resonance frequency. Depending on the operating frequency, its impedance changes, resulting in a reduced impedance at the tuned resonant frequency that allows the harmonic current to be absorbed and sent to ground. Equation (1) provides the overall filter impedance at kth harmonic order (Z_k_)^33^:

1 $${\text{Z}}_{\text{k}}={\text{R}}_{\text{k}}+\text{j}\left(\upomega {\text{L}}_{\text{k}}-\frac{1}{\upomega {\text{C}}_{\text{k}}}\right)$$

where;

${\text{R}}_{\text{k}}$ is the filter resistance at kth harmonic order. $\upomega {\text{L}}_{\text{k}}$ is the filter inductive reactance at kth harmonic order. $\frac{1}{\upomega {\text{C}}_{\text{k}}}$ is the filter capacitive reactance at kth harmonic order. At the resonance frequency, the inductive reactance and capacitive reactance equal each other. Therefore, Z_k_ = R_k_ as shown in Fig. 2.

A single-tuned filter is designed based on three parameters^32^:

i. Tuned frequency or harmonic current order that is required to be blocked.
ii. Quality factor that is the ratio of the inductance (or capacitance) to resistance at the resonant frequency and it determines the sharpness of tuning.
iii. Capacitance value.
iv. System voltage level and the fundamental frequency.

The tuned resonance frequency f_k_ of a specific k^th^ filter and the quality factor are represented by Eq. (2) as follows^34^:

2 $${\text{f}}_{{\text{k}}} = \frac{1}{{2{\uppi }\sqrt {{\text{L}}_{{\text{k}}} {\text{C}}_{{\text{k}}} } }} \left( {Hz} \right)$$

where, L_k_ and C_k_ are the filter matching values for capacitive and inductance, accordingly, quality factor is represented by Eq. (3) as follows^4,34^:

3 $$Quality factor=\frac{X}{R}$$

When x is reactance and R is resistance.

The capacitance initial value (C_initial_) could be determined by the reactive energy associated to the filter (Q_k_) according to fundamental voltage (V_1_) and fundamental angular frequency (ω_1_) of the system by Eq. (4) as follows^32^:

4 $${\text{C}}_{\text{initial}}=\frac{{\text{Q}}_{\text{k}}}{{\upomega }_{1}{\text{V}}_{1}} \left(\text{farad}\right)$$

## System design and components

**Fig. 3:**
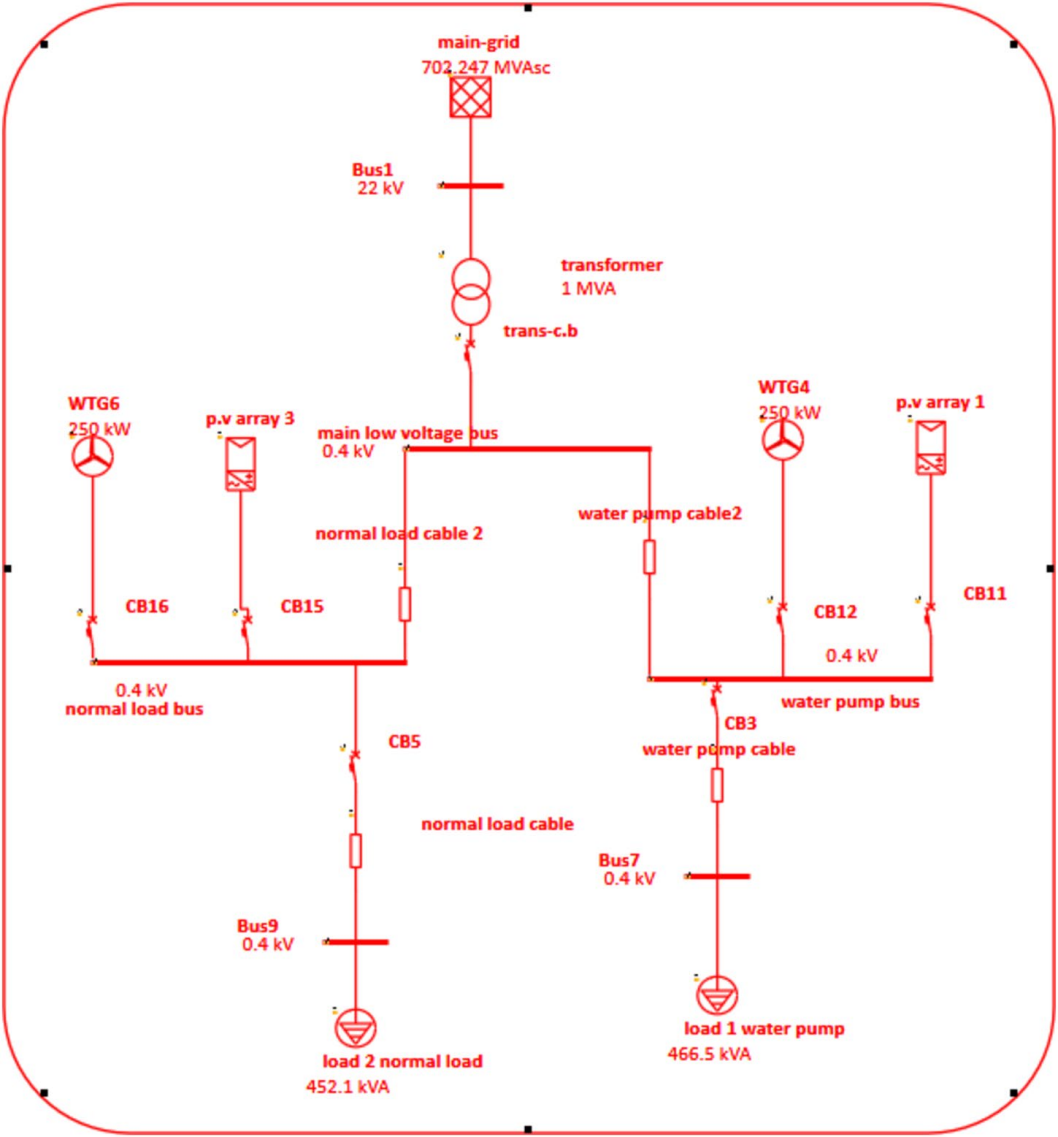
Microgrid organization.

Figure 3 illustrates the components of the MG organization, which include a PV array, an inverter, loads, a utility grid, and a wind energy resource.

### PV panel model

Photovoltaic (PV) arrays and modules, which are made of the primary solar cell components of the PV system, make up a PV panel. A PV module is made up of PV cells coupled in both series and parallel arrangements. A PV array is created by combining many PV modules. This study examines the utilization of a 250 KW PV array is modeled by E-Tap program^35^.

### Wind energy model

The wind’s dynamic kinetic energy is initially converted into rotational energy by Wind Turbines (WT). This energy is then synchronized with the turbine and generator’s speed through the use of a gearbox. The generator transforms the kinetic energy produced by the turbine into electrical energy. The WT has a power rating of 250 kilowatts^36^.

### Battery energy storage system model

BESS is a device that uses batteries to store electric power under optimal conditions and supply it during power shortages. The predominant type of BESS used to optimize the performance of PV arrays is lead-acid batteries. The battery’s charging and discharging processes are regulated based on its State of Charge (SOC), with discharging occurring at 80% SOC and charging occurring at 20% SOC^37,38^.

### Flexible electrical loads

**Fig. 4:**
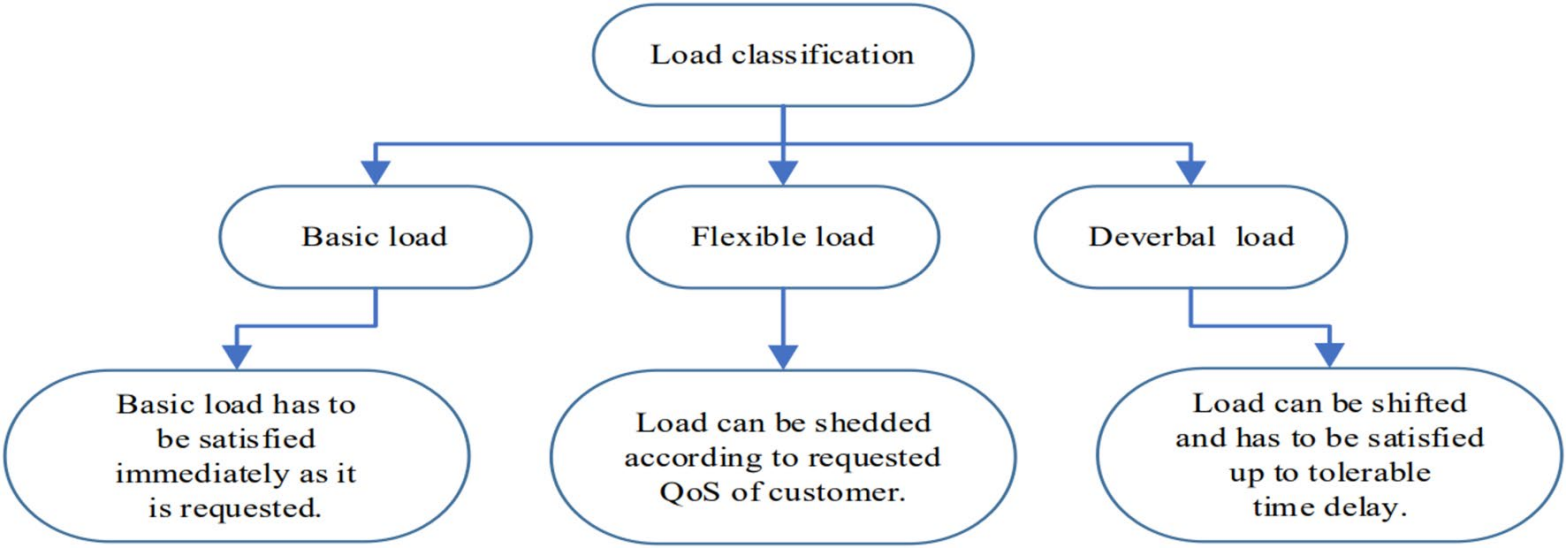
Classification of electrical load.

In modern MGs, load flexibility is beneficial in many aspects^39^. Generally, loads can be classified into three categories, as depicted in Fig. 4.

### Summary

Finaly due to the increase in load and distance from the source, there was a need to increase electrical capacity to match the load and overcome power quality issues. Renewable energy sources were added to increase the electrical power generated to overcome the increase in load and to reduce dependence on the electrical grid, and renewable sources were added near the loads to overcome some power quality issues such as increased voltage drop and fading, and in this simple way, many power quality issues were overcome.

After the addition of renewable energy sources, we had another issue of harmonics, and to overcome this issue, a harmonic filter was added to the grid to overcome the harmonics resulting from the use of renewable energy components.

## Simulation results

**Case 1**: by making a load flow study for the grid without adding any RES.

**Fig. 5:**
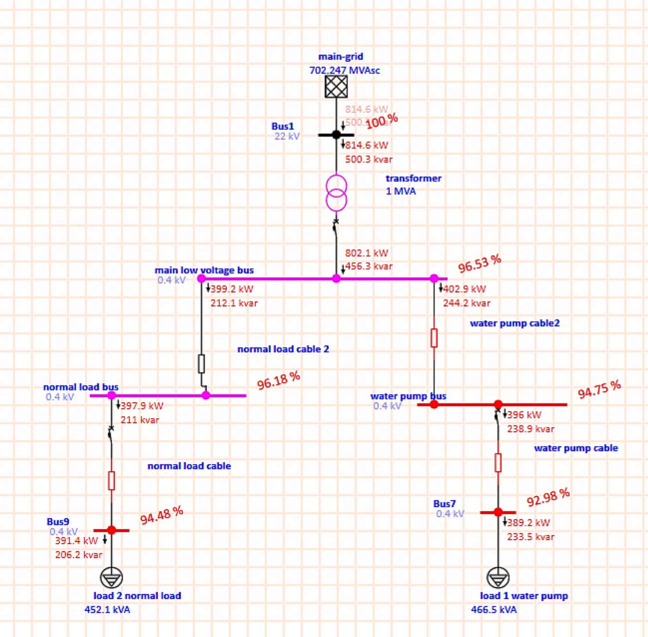
Load flow analysis for grid.

As shown in Fig. 5 after applying load flow analysis on the network before MG installation by using the Electrical Transient Analyzer Program (ETAP) program can see the following:

- High voltage drops and under voltage.
- Transformer is overloaded.
- Low power quality.
- Less efficient system.
- Non-reliable system.

From ETAP report; Table 1 shows that, the load and consumption and power losses, the power losses of active and reactive is high at this system thus the total consumption is high because the consumption equal load plus loss.

**Table 1:** Load, consumption and loss for the overall system.

Load-MW	0.781
Load-MVar	0.44
Consumption -MW	0.815
Consumption -MVar	0.5
Loss-MW	0.0341
Loss-MVar	0.0606

From ETAP report; Table 2 shows that, the voltage at bus1, its moderate and equal nominal voltage, but the voltage at other busses its decrease than nominal voltage and the voltage drop at the load buses is high.

**Table 2:** Voltage drop for every bus.

Bus ID	Nominal KV	Type	Voltage%
Bus1	22	SWNG	100
Bus7	0.4	Load	92.98
Bus9	0.4	Load	94.48
Main low voltage bus	0.4	Load	96.53
Normal load bus	0.4	Load	96.18
Water pump bus	0.4	Load	94.75

From ETAP report; Table 3 shows that, the flow of active power KW and reactive power KVar and also show the losses and power factor, from this table noted that the power factor is low at all busses and the losses is high for every cable and total losses at transformer is very high.

**Table 3:** Active and reactive power flow and losses and % PF for every bus.

ID	Type	KW Flow	KVar Flow	% PF	KW Losses	KVar Losses
Normal load cable	Cable	397.9	211	88.34	6.45	4.82
Normal load cable 2	Cable	397.9	211	88.34	1.29	1.1
Transformer	Transf	814.6	500.3	85.22	12.55	43.94
Water pump cable	Cable	389.2	233.5	85.75	6.89	5.36
Water pump cable2	Cable	396	238.9	85.63	6.89	5.36

From ETAP report; Table 4 shows that, the state of the bus and cable, all buses and cable have problems such as under voltage and overloaded.

**Table 4:** State of the important bus.

Device ID	Type	Condition
Bus7	Bus	Under voltage
Bus9	Bus	Under voltage
Normal load cable	Cable	Overload
Water pump bus	Bus	Under voltage
Water pump cable	Cable	Overload
Water pump cable2	Cable	Overload

To overcome that problems, some steps must be taken, including the depreciation of loads or the use of other sources of energy to reduce loads from the traditional source and improve power quality. In order to address the aforementioned issue, the following measures were implemented.

**Fig. 6:**
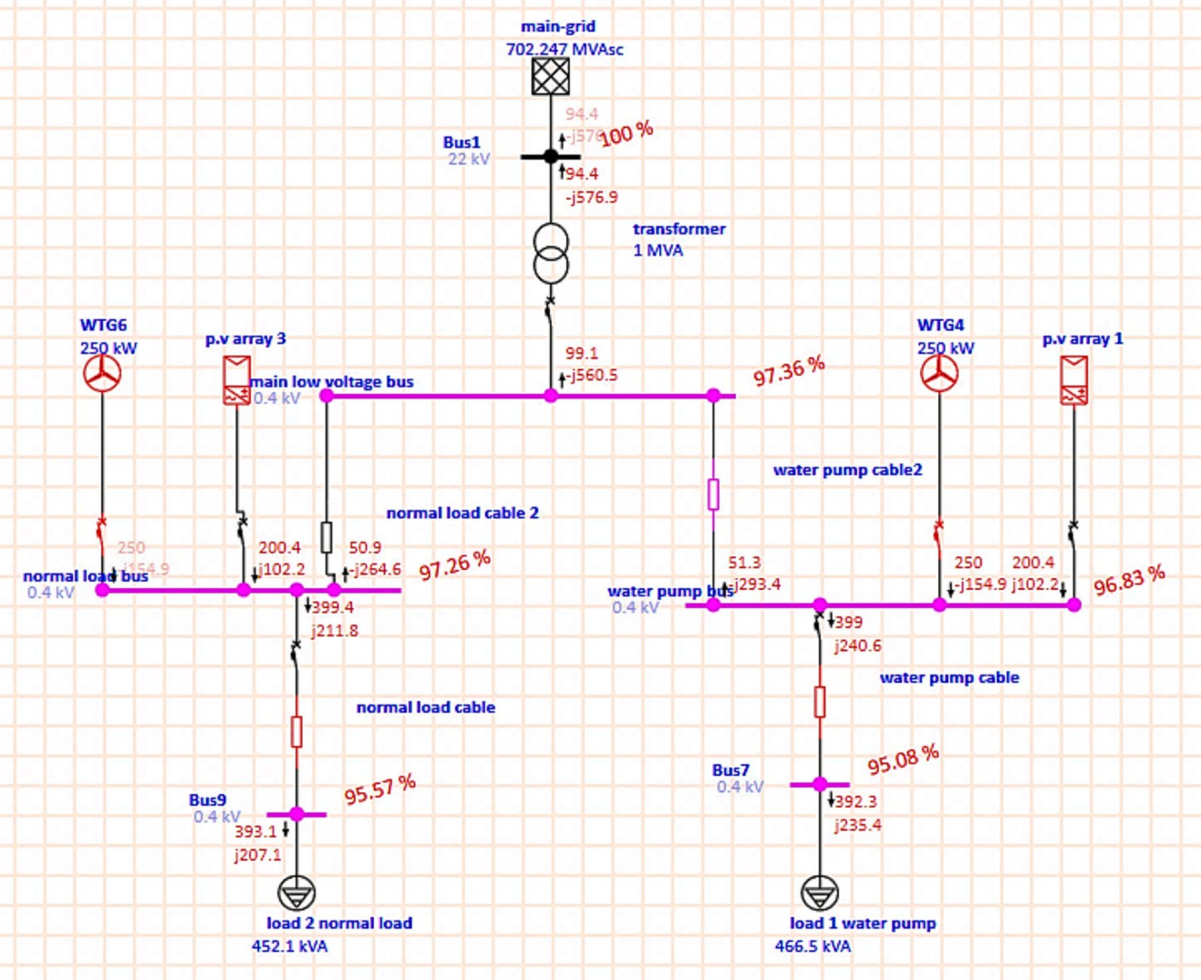
Microgrid with RES.

The revised system architecture calls for using the electrical energy generated by PV and WT to supplement grid supply in order to fulfill demand and prevent the aforementioned issue. Study the load flow analysis by using ETAP program. As shown in Fig. 6, the MG after adding RES to the grid, the wind turbine is an induction generator that injects active power and absorbs reactive power, which causes a voltage drop at the MG and poor power quality; voltage and power quality can be modified to inject active power and not absorb reactive power.

**Fig. 7:**
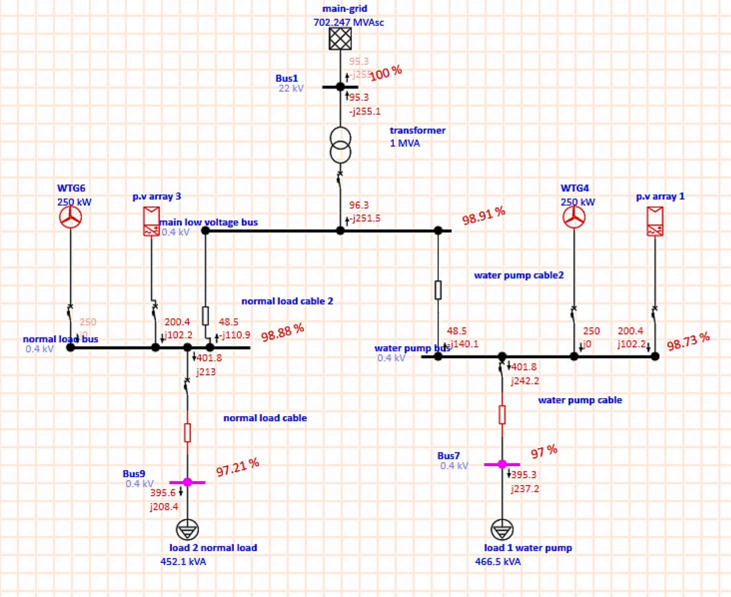
Microgrid with RES after modification.

As shown in Fig. 7, after modifying the wind turbine at the simulation program by selecting wind turbine as voltage control wind turbine to maintained voltage and absorb zero reactive power, the voltage is improved, power quality is improved, reactive power is reduced, losses and consumption are reduced, and active and reactive power is also injected into the main grid.

From ETAP report; Table 5 shows that, the power losses are reduced, consumption is reduced and there is a generation from RES sufficient to meet the load and the active and reactive power is injected to grid.

**Table 5:** Load and generation, loss for overall system.

Load-MW	0.791
Load-MVar	0.446
Generation-MW	0.805
Generation-MVar	0.46
Loss-MW	0.0145
Loss-MVar	0.0143

From the ETAP report, Tables 6, 7, and 8 show that the voltage drop is reduced, the voltage profile is improved, and the consumption of reactive power is reduced, and at the specific buses, reactive power is injected into the grid, and the power factor is improved, and the load and generation at all buses.

**Table 6.** Percentage of voltage for every bus.

Bus ID	Nominal KV	Type	%Voltage
Bus1	22	SWNG	100
Bus7	0.4	Load	97
Bus9	0.4	Load	97.21
Main low voltage bus	0.4	Load	98.91
Normal load bus	0.4	Gen	98.88
Water pump bus	0.4	Gen	98.73

**Table 7.** Active and reactive power flow and losses and p.f for every bus.

ID	Type	KW Flow	KVar Flow	% PF	KW Losses	KVar Losses
Normal load cable	Cable	401.8	213	88.35	6.22	4.65
Normal load cable 2	Cable	48.53	− 110.9	− 40.09	0.0884	0.0747
Transformer	Transf	95.32	− 255.1	− 35	1.02	3.57
Water pump cable	Cable	395.3	237.2	85.75	6.53	5.08
Water pump cable2	Cable	48.55	− 140.1	-32.75	0.652	0.506

From the ETAP report, Table 8 shows that the MG with RES injects active power into the grid.

**Table 8.** Load and generation at every type.

ID	Type	Rated KV	MW	MVar	% Generation
Main-grid	Power Grid	22	− 0.0953	0.255	
PV array 1	PV and Inverter	0.4	0.2	0.102	101.2
PV array 3	PV and Inverter	0.4	0.2	0.102	101.1
WTG4	Wind Turbine	0.4	0.25	0	100
WTG6	Wind Turbine	0.4	0.25	0	100

**Case 2**: by making a harmonic load flow study for the MG after adding renewable energy sources.

**Fig. 8:**
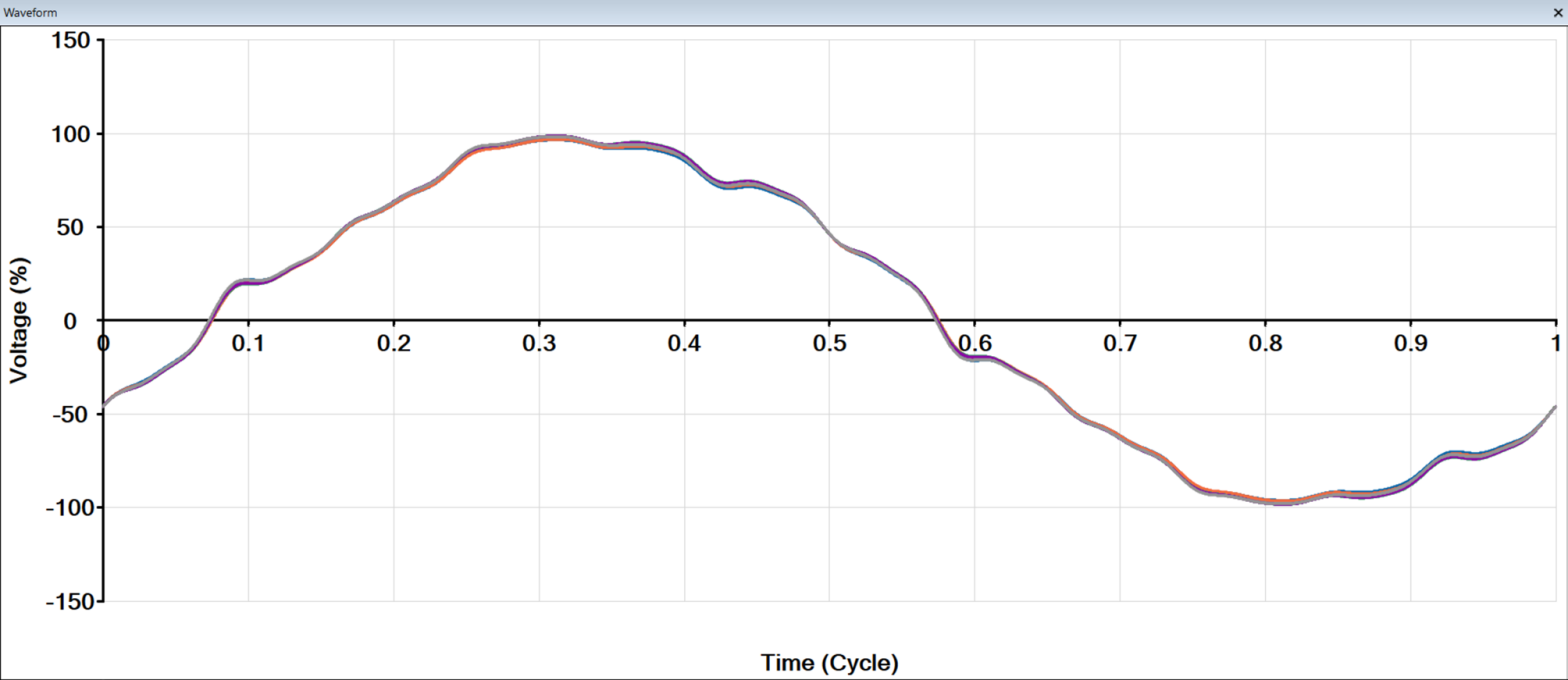
Shape of wave form after adding RES.

Figure 8 shows the ship of waves from all buses and the fundamental wave. After adding RES, the cause of this distortion is harmonics.

**Fig. 9:**
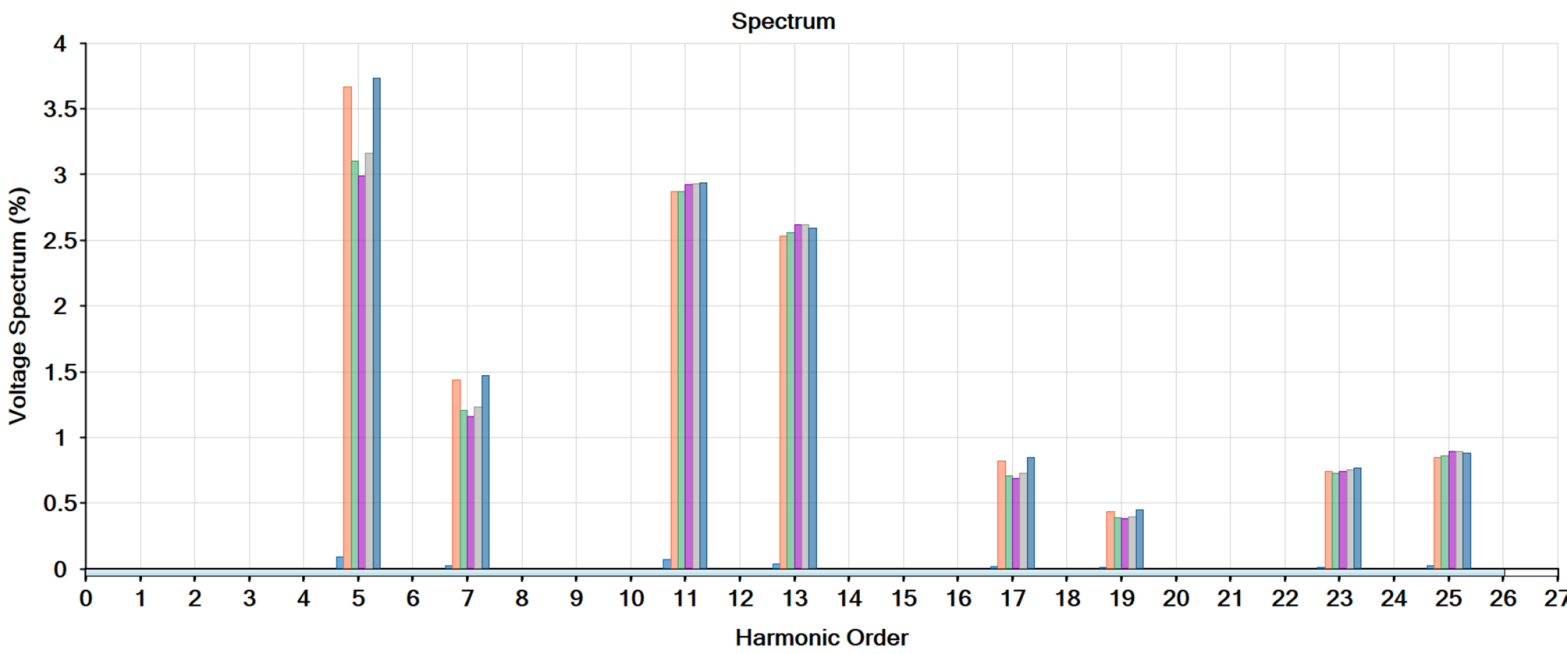
Frquency scan.

By frequency scanning the percentage of the voltage spectrum at every harmonic order at every bus after adding RES, as shown in Fig. 9. The 5th, 7th and 11th, 13th harmonics have a significant effect on the system and their magnitude is high, so it is necessary to install a harmonic filter.

**Fig. 10:**
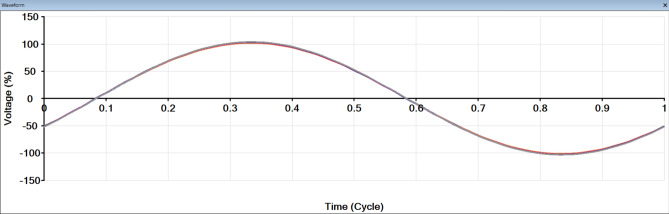
Shape of waveforms after adding harmonic filter.

**Fig. 11:**
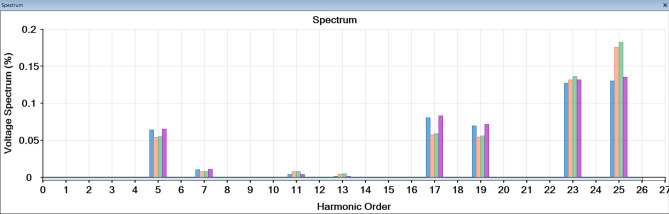
Frequency scan after adding harmonic filter.

To avoid this problem, a harmonic filter is added to the system to remove some of the affected harmonics. Figure 10 shows the shape of the waves for all buses after adding a harmonic filter. It was found that the distortion was removed, the wave became smooth, and the 5th, 7th and 11th, 13th harmonics were removed, and THD is reduced, as shown in Fig. 11.

**Fig. 12:**
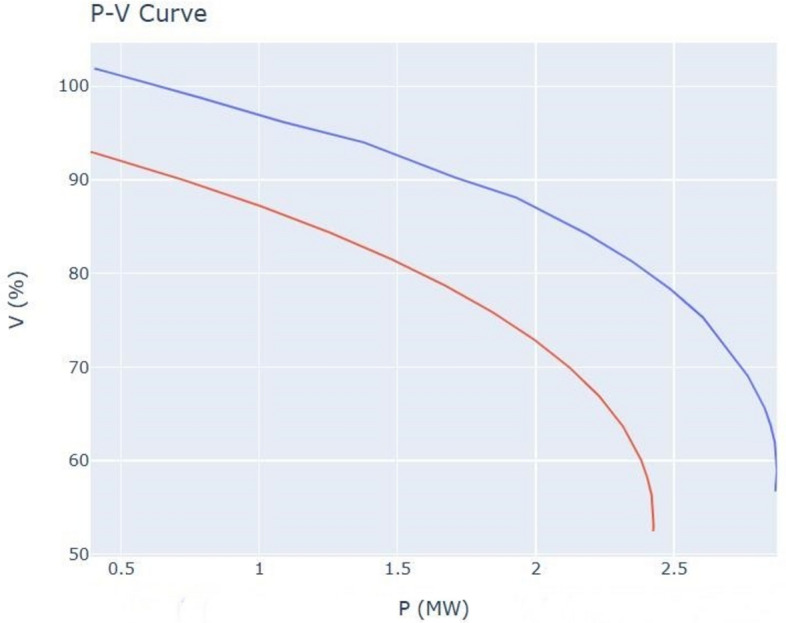
P–V curve.

When the active power is raised, the amount of voltage is reduced, and thus voltage drop and under voltage occurs as shown at P–V curve at Fig. 12. The red curve represents the system before adding MG and blue curve represent the system after adding it. The system after adding RES is more stable than the system before adding it.

## Conclusion

This paper addresses several power quality concerns in MGs and clarifies the power quality issues in both AC and DC MGs. The proliferation in distributed Power Source has exacerbated power quality problems and is a significant challenge for both large consumers and utility companies. Also, this paper looks at power quality issues and how to improve them. The presence of harmonics has been observed to have deleterious consequences on the MG. Also, this paper presents various power quality issues that arise in a stable state, specifically voltage drop, harmonic distortion, power losses, and phase unbalance. This will be achieved by adjusting the demand level of specific electrical devices in the MG, adding other sources to the grid near the load center, such as renewable energy sources, and studying the impact of these sources on the performance and behavior of the grid. This study will examine these problems both before and after the integration of RES to MG. A real-life scenario with the aforementioned issues has been selected for analysis. The results show that the voltage profile and voltage distortion, production, and the consumption and losses, harmonics in the MG and study this result before adding RES and after adding it and comparing between result of MG and traditional grid.

### Limitation and future work

One of the study’s shortcomings is its narrow focus on power quality elements, while another study aims to reduce consumption voltage drop and improve power quality through the use of renewable energy sources.

The diversity and accessibility of renewable energy sources could enhance research and expand the range of viable economic options, potentially making the suggested solution applicable in other contexts. This work’s limitations stem from the fact that it ignores crucial issues like protection and data processing.

Furthermore, it doesn’t handle hybrid cars, which can represent the source or the load depending on the situation thanks to a battery. Future articles could delve deeper into this significant topic. The following features of cooperative MG control may be included in order to enable supplemental grid services. Adaptability; dependability; security; and robustness.

## Data Availability

Data generated or analyzed during this study is included in this article.

## References

[1] J. Charles Rajesh Kumar and M. A. Majid, Renewable energy for sustainable development in India: Current status, future prospects, challenges, employment, and investment opportunities, *Energy. Sustain. Soc.*, 10.1186/s13705-019-0232-1, (2020).

[2] S. L. Chartier, V. K. Venkiteswaran, S. S. Rangarajan, E. R. Collins, and T. Senjyu, Microgrid Emergence, Integration, and Influence on the Future Energy Generation Equilibrium—*Review, Electron.*, (2022).

[3] V. Reshmi, M. Ebenezer, Power Quality Enhancement Techniques in Microgrid-A Review. In 2023 International Conference on Control, *Commun. Computing (ICCC)*, (2023).

[4] Wessam A. Hafez, Montaser Abd El Sattar, Ali H. Kasem Alaboudy and Adel A. Elbaset, Power Quality Issues of Grid Connected Wind Energy System Focus on DFIG and Various Control Techniques of Active Harmonic Filter: A review, 2019 21st International Middle East Power Systems Conference (MEPCON), Tanta University, Egypt, (2019).

[5] Rezkallah, M., Chandra, A., Singh, B. & Singh, S. microgrid: configuration, control and applications. *IEEE Trans. Smart Grid* **10**(2), 1290–1302 (2017).

[6] Wang, X. et al. Real World scale deployment of hydrogen-integrated microgrid: design and control. *IEEE Trans. Sustain. Energy* **15**(4), 2380–2392 (2024).

[7] Patel, Astha Bharat, and Rajasekharareddy Chilipi. Current Control Strategy using Sinusoidal Signal Integrators for Harmonic Elimination in a Standalone Microgird. 2023 IEEE International Conference on Energy Technologies for Future Grids (ETFG). IEEE, (2023).

[8] M. Abdelsattar, Mohamed A. Ismeil, Mohamed M. Aly, Salah SabeR Abu-elwfa, Energy Management of Microgrid With Renewable Energy Sources: A Case Study in Hurghada Egypt, IEEE ACCESS, Volume 12, pp. 19500- 19509, 2024.

[9] Salem, A. E., Arafah, S. H. & Salim, O. M. Power quality enhancement of grid-islanded parallel microsources using new optimized cascaded level control scheme. *Int. J. Electr. Power Energy Syst.* **140**, 108063 (2022).

[10] B. Nanda, R. K. Jena, and B.P. Muni, A Review Report On Power Quality Improvement Techniques In Ac / Dc Hybrid Microgrid. *J. Pharm. Negative Results.* (2022).

[11] G. S.Chawda, A. G. Shaik, M.Shaik, S.Padmanaban, J. B.,Holm-Nielsen, O. P. Mahela, & Kaliannan, P., Comprehensive review on detection and classification of power quality disturbances in utility grid with renewable energy penetration. IEEE Access, (2020).

[12] Al-Shetwi, Ali Q. "Sustainable development of renewable energy integrated power sector: Trends, environmental impacts, and recent challenges. *Sci. Total Environ.*, 822, p.153645, (2022). 10.1016/j.scitotenv.2022.153645 35124039

[13] M. Abdelsattar, Mohamed A. Ismeil, Mohamed M. A. Azim Zayed, Ahmed Abdelmoety, Ahmed Emad-Eldeen, Assessing Machine Learning Approaches for Photovoltaic Energy Prediction in Sustainable Energy Systems,” IEEE ACCESS, Volume 12, pp. 107599- 1076, Aug. 2024.

[14] Sepasi, S., Talichet, C. & Pramanik, A. S. Power Quality in Microgrids: A Critical Review of Fundamentals, Standards, and Case Studies. *IEEE Access* **11**, 108493–108531. 10.1109/ACCESS.2023.3321301 (2023).

[15] He, J., Liang, B., Li, Y. W. & Wang, C. Simultaneous microgrid voltage and current harmonics compensation using coordinated control of dual-interfacing converters. *IEEE Trans. Power Electron.* **32**(4), 2647–2660 (2017).

[16] J. Malar, Sheeba Jeba, A. Bisharathu Beevi, and M. Jayaraju. Efficient power flow management in hybrid renewable energy systems. *IETE J. Res.* (2023).

[17] Mendes, T. M. et al. PLL based method for supraharmonics emission assessment. *IEEE Trans. Power Delivery* **37**(4), 2610–2620 (2021).

[18] Adineh, Behrooz, et al. Review of harmonic mitigation methods in microgrid: From a hierarchical control perspective. *IEEE J. Emerging Selected Topics Power Electron.*, 9, 3044–3060, (2020).

[19] Mahfuz-Ur-Rahman, A. M., Islam, M. R., Muttaqi, K. M., & Sutanto, D. An effective energy management with advanced converter and control for a PV-battery storage based microgrid to improve energy resiliency. *IEEE Trans. Industry Appl.* (2021).

[20] Aladesanmi, E. J. & Ogudo, K. Microgrids Overview and Performance Evaluation on Low-voltage Distribution. *Network*10.20944/preprints202309.1389.v1 (2023).

[21] Ertek, G. & Kailas, L. Analyzing a decade of wind turbine accident news with topic modeling. *Sustainability* **13**(22), 12757 (2021).

[22] VAN DEN BROM, Helko E., et al. Power Quality Measurement Results for a Configurable Urban Low-Voltage DC Microgrid. *Energies*, (2023).

[23] Nejabatkhah, Farzam, Yun Wei Li, and Hao Tian. Power quality control of smart hybrid AC/DC microgrids: An overview. *IEEE Access.*, (2019).

[24] Afonso, L.Joao, et al. "A Review on Power Electronics Technologies for Power Quality Improvement. *Energies*, 2021).

[25] Backhaus, S.N., Swift, G.W., Chatzivasileiadis, S., Tschudi, W., Glover, S., Starke, M., Wang, J., Yue, M., Hammerstrom, D. DC microgrids scoping study. Estimate of Technical and Economic Benefits. Los Alamos National Lab, (LANL), Los Alamos NM (United States), (2015).

[26] Blaz, Michael, et al. Setup for testing energy meters with disturbed DC signals occuring in DC charging stations. 2023 IEEE 13th International Workshop on Applied Measurements for Power Systems (AMPS). IEEE, (2023).

[27] Rai, I., Ravishankar, S. & Anand, R. Review of DC Microgrid system with Various Power Quality Issues in “Real Time Operation of DC Microgrid Connected System. *Majlesi J. Mechatron. Syst.* **8**, 35–44 (2020).

[28] K.Jithin, et al. A review on challenges in dc microgrid planning and implementation. *J. Modern Power Syst. Clean Energy*, (2022).

[29] Khezri, R., Mahmoudi, A. & Aki, H. Optimal planning of solar photovoltaic and battery storage systems for grid-connected residential sector: Review, challenges and new perspectives. *Renew. Sustain. Energy Rev.*10.1016/j.rser.2021.111763 (2022).

[30] Bollen, M. H. J. et al. Power quality concerns in implementing smart distribution-grid applications. *IEEE Trans. Smart Grid*10.1109/TSG.2016.2596788 (2016).

[31] Rönnberg, S. K. et al. On waveform distortion in the frequency range of 2 kHz–150 kHz—Review and research challenges. *Electric Power Syst. Res.*10.1016/j.epsr.2017.04.032 (2017).

[32] Melo, I. D., Pereira, J. L. R., Variz, A. M. & Ribeiro, P. F. Allocation and sizing of single tuned passive filters in three-phase distribution systems for power quality improvement. *Electric Power Syst. Res.* **180**, 106128 (2020).

[33] Eroğlu, Hasan, et al. Harmonic problems in renewable and sustainable energy systems: A comprehensive review. Sustain. Energy Technol. Assessments, (2021).

[34] Abd, A. M., El-Hameid, A. A., Elbaset, M. E. & Abdelsattar, M. *Enhancement of Grid-Connected Photovoltaic Systems Using Artificial Intelligence* (Springer, 2023).

[35] Sattar, M. A. E., Abd, A. M., Hamed, El., Elbaset, A. A. & Ebeed, M. Optimal sites and sizes of DSTATCOM and PV based DG using Manta Ray Foraging Optimizer. *Inter. J. Innovative Eng. Manag. Res.* **10**(08), 71–79 (2021).

[36] Montaser Abd El Sattar, Wessam Arafa Hafez, Adel A. Elbaset, and Ali H. Kasem Alaboudy, Economic Valuation of Electrical Wind Energy in Egypt Based on Levelized Cost of Energy, *Inter. J. Renew. Energy Res.*, (2020).

[37] Nazir, M. S., Wang, Y., Bilal, M. & Abdalla, A. N. Wind Energy, Its Application, Challenges, and Potential Environmental Impact. In *Handbook of Climate Change Mitigation and Adaptation* (ed. Chen, W.-Y.) (Springer, 2021).

[38] Kasem, A. H., Alaboudy, H. A., Mahmoud, A. A. & Elbaset, M. A. A Case Study on the LVRT Capability of an Egyptian Electrical Grid Linked to the Al-Zafarana Wind Park using Series Resistor. *Inter. J. Renew. Energy R.* **13**(1), 36–48 (2023).

[39] Xu, X., Bishop, M., Oikarinen, D. G. & Hao, C. Application and modeling of battery energy storage in power systems. *CSEE J. Power Energy Syst.* **2**, 82–90 (2016).

